# Dopamine, time perception, and future time perspective

**DOI:** 10.1007/s00213-018-4971-z

**Published:** 2018-07-19

**Authors:** Jennifer M. Mitchell, Dawn Weinstein, Taylor Vega, Andrew S. Kayser

**Affiliations:** 1 Department of Neurology, University of California, San Francisco, 675 Nelson Rising Lane, San Francisco, CA 94143 USA; 2 Department of Psychiatry, University of California, San Francisco, San Francisco, USA; 3 Department of Neurology, VA Northern California Health Care System, Martinez, USA

**Keywords:** Dopamine, Time perspective, Time perception, Impulsivity, Treatment

## Abstract

**Rationale:**

Impairment in time perception, a critical component of decision-making, represents a risk factor for psychiatric conditions including substance abuse. A therapeutic that ameliorates this impairment could be advantageous in the treatment of impulsivity and decision-making disorders.

**Objectives:**

Here we hypothesize that the catechol-*O*-methyltransferase (COMT) inhibitor tolcapone, which increases dopamine tone in frontal cortex (Ceravolo et al Synapse 43:201–207, 2002), improves time perception, with predictive behavioral, genetic, and neurobiological components.

**Methods:**

Subjects (*n* = 66) completed a duration estimation task and other behavioral testing in each of two sessions after receiving a single oral dose of tolcapone (200 mg) or placebo in randomized, double-blind, counterbalanced, crossover fashion. Resting state fMRI data were obtained in a subset of subjects (*n* = 40). Subjects were also genotyped for the COMT (rs4680) polymorphism.

**Results:**

Time perception was significantly improved across four proximal time points ranging from 5 to 60 s (*T*(524) = 2.04, *p* = 0.042). The degree of this improvement positively correlated with subjective measures of stress, depression, and alcohol consumption and was most robust in carriers of the COMT Val158 allele. Using seed regions defined by a previous meta-analysis (Wiener et al Neuroimage 49:1728–1740, 2010), we found not only that a connection from right inferior frontal gyrus (RIFG) to right putamen decreases in strength on tolcapone versus placebo (*p* < 0.05, corrected), but also that the strength of this decrease correlates inversely with the increase in duration estimation on tolcapone versus placebo (*r* = − 0.37, *p* = 0.02).

**Conclusions:**

Compressed time perception can be ameliorated by administration of tolcapone. Additional studies should be conducted to determine whether COMT inhibitors may be effective in treating decision-making disorders and addictive behaviors.

**Electronic supplementary material:**

The online version of this article (10.1007/s00213-018-4971-z) contains supplementary material, which is available to authorized users.

## Introduction

Our subjective experience of time shapes our decision-making. If a task is perceived as taking a lengthy time to complete, some will find it hard to wait while others will be unperturbed. As anyone who has ever taken a long car trip knows, individuals have different thresholds for deciding when to ask, “Are we there yet?” Similarly, many find it difficult to persevere in the face of unanticipated challenges—i.e., to continue despite obstacles and setbacks and to avoid making impulsive, ultimately disadvantageous decisions. Intriguingly, this type of temporal impulsivity also contributes to the propensity to develop problems with alcohol, drugs, gambling, overspending, and overeating (Hamilton et al. 2015; London 2016) and to the deleterious behavioral consequences of neurodegenerative diseases such as frontotemporal dementia (Bertoux et al. 2015). Expanding time horizon could potentially enable vulnerable individuals to wait longer for appropriate rewards and thereby improve many decision-making disorders.

Consistent with the important role of dopamine in reward-based decision-making, several clinical conditions involving dopaminergic dysfunction are characterized by impaired time perception, such as stimulant abuse (Wittmann et al. 2007) and Parkinson’s disease (Jones et al. 2008), suggesting that the dopamine system could be fundamentally involved in temporal decision-making (Wiener et al. 2011). Indeed, a number of investigators have hypothesized that individuals who are hypo-dopaminergic specifically in frontal regions are most susceptible to difficulties with accelerated time perception (Blum et al. 2007; Gold et al. 2015) and by extension are more likely to develop problems with alcohol and drug abuse (Conner et al. 2010). In keeping with this idea, variations in the dopamine-metabolizing enzyme catechol-*O*-methyltransferase (COMT), as well as in other dopaminergic genes such as DRD2, which encodes the dopamine D2 receptor, have been linked to behavioral differences in time perception in healthy controls (Balci et al. 2013; Wiener et al. 2011).

Similarly, differences in time perception are likely to have a specific neural correlate. A recent meta-analysis of time perception across a variety of tasks and studies (Wiener et al. 2010) demonstrated associated neural activity within two prefrontal brain regions, the right inferior frontal gyrus (RIFG) and the supplemental motor area (SMA). Moreover, greater activation in lateral prefrontal and striatal regions correlates with improved time perception (Wiener et al. 2014) and depletion of dopamine precursors leads to impairments in time perception as well as decreased timing-specific BOLD signal in the SMA and putamen (Coull et al. 2012). These studies suggest that increasing dopamine in prefrontal cortex may correspondingly improve time perception, and that this improvement may be mediated by changes in top-down control over behavior (i.e., via altered connectivity between relevant prefrontal regions and the striatum). Because the FDA-approved COMT inhibitor tolcapone acts to preferentially augment dopamine tone in frontal regions of the brain (Doudet et al. 1997), this medication may be useful in assessing this hypothesis, especially as previous work has shown that tolcapone can improve behavioral self-regulation and, correspondingly, alter the magnitude of correlations between frontal and striatal brain areas (Kayser et al. 2012; Kayser et al. 2017).

Here we investigate whether a single oral dose of the COMT inhibitor tolcapone alters time perception, and whether a relationship exists between the effects of tolcapone on measures of time perception, time perspective, and future orientation. We additionally assess whether the effects of tolcapone on measures of time perception are modified by the COMT Val158Met genotype, a functional polymorphism in the catecholamine-O-methyltransferase (COMT) gene that impacts frontal cognitive function (Apud et al. 2007; Egan et al. 2001). As there are well-established relationships between hazardous alcohol use, behavioral impulsivity, and mood disorders (Dick et al. 2010; Jentsch and Taylor 1999; Koob and Le Moal 1997), we also assess the degree to which these behavioral measures correlate with the magnitude of the tolcapone effect on time perception. Finally, we test whether tolcapone leads to changes in resting state connectivity in frontostriatal circuits associated with time perception. Our overarching aim is to take the first steps to determine whether a COMT inhibitor could be of therapeutic benefit to populations suffering from psychiatric conditions that affect decision-making.

## Materials and methods

### Subjects

All subjects (*n* = 66; 33 females) gave written informed consent in accordance with the Human Research Protection Program (HRPP) at the University of California, San Francisco, University of California, Berkeley, and Department of Defense Human Research Protection Office (HRPO). Subjects underwent a medical history, physical examination, and blood draw to evaluate liver enzyme levels prior to completing a number of screening questionnaires, including the Stanford Time Perspective Inventory (STPI), the Alcohol Use Disorders Identification Test (AUDIT), the Depression, Anxiety, and Stress Scale (DASS), the Obsessive Compulsive Drinking Scale (OCDS), and the Barratt Impulsiveness Scale (BIS). Subjects were excluded for recent psychoactive drug use, clinically significant medical or psychiatric illness, liver function tests ≥ 3 times the normal upper limit, a history of liver disease, regular use of any drugs with either dopaminergic actions or contraindications for use with tolcapone, and pregnancy or breastfeeding (females). Subjects were additionally screened for MRI criteria and were excluded for any conditions that could render MRI unsafe. Time perception data were collected as part of larger neuropharmacology studies conducted at the UCB Brain Imaging Center (below). None of the time perception data contained herein were used in conjunction with outcome measures from those studies.

Subjects were administered either 200 mg tolcapone or placebo in a randomized, double-blind, counterbalanced, crossover design. This dose of tolcapone was selected based on our previously published findings indicating that a single 200 mg tolcapone dose produces reliable behavioral results without significant or consistent side effects (Cameron et al. 2018; Kayser et al. 2012; Kayser et al. 2015; Kayser et al. 2017;Saez et al. 2015). Order was assigned using standard random allocation sequence-generating software by a study investigator who did not otherwise have contact with subjects. Subjects and all study staff who had contact with subjects were blinded to the intervention: medication bottles were labeled with a unique letter for each drug condition and did not otherwise contain identifiable information. The placebo and tolcapone capsules were visually identical and were compounded by a licensed compounding pharmacist together with 25 mg of the B vitamin riboflavin in order to mask any urine discoloration produced by tolcapone. Between 2 and 3 h after drug administration (i.e., during presumptive peak tolcapone levels (Deleu et al. 2002; Jorga et al. 2000)), subjects completed a computerized time perception task in which they were asked to make a key press after they perceived that specific durations of time had passed (5, 15, 30, and 60 s). The four time intervals were independently presented. Subjects were monitored for counting strategies but were not explicitly told not to count.

To ensure that any effects of tolcapone were not related to lower level effects on motor responding, subjects also completed a control task assessing reaction time (RT) (Kayser et al. 2012; Wittmann and D'Esposito 2015). In brief, subjects were required to press the space bar as quickly as possible following the brief appearance of a fixation cross in the center of an otherwise blank screen. To avoid allowing subjects to predict the appearance of the fixation cross, the inter-stimulus interval was jittered between 2000 and 5000 ms, with intervals drawn from a uniform distribution discretized into 50 ms bins. To reduce the influence of attentional lapses, we cued subjects with a tone presented between 150 and 750 ms prior to the appearance of the fixation cross, with the interval drawn from a uniform distribution discretized into 150 ms bins. Subjects completed 25 trials over approximately 2 min just prior to study drug administration and again 3–4 h following drug administration. The difference between the mean RTs for each of these instances of the task was compared across drug and placebo sessions.

At the conclusion of the final study visit, subjects were asked which visit they believed they had received study drug and which they had received placebo. Subjects performed at chance when asked to guess which drug cycle they were on, suggesting that the subjective effects of tolcapone were not largely distinguishable from placebo.

To analyze behavior, we pursued two approaches. For one set of behavioral analyses, we directly evaluated the absolute difference in seconds between the actual and the desired durations (inaccuracy = **Δ***t*). This approach was particularly useful for comparison of temporal task performance with behavioral questionnaires evaluating drug and alcohol use, for which we focused on **Δ***t* values obtained for the 60 s duration, as previous data indicate that inaccuracy increases over time (Buhusi and Meck 2005). Because such behavioral questionnaires capture broader time frames than those used here for time perception testing, we hypothesized that using the longest time perception measurement would maximize the opportunity to identify a relationship between the shorter interval effects tested here and the longer interval effects assessed by the questionnaires.

For the other set of analyses, we divided the absolute difference in seconds between the actual and desired durations for the duration estimation task by the desired interval in seconds (inaccuracy = **Δ***t*/*t*). This ratio approach, akin to evaluating the Weber fraction (Wittmann et al. 2007), reflects potentially fundamental principles of sensory perception (Norwich 1987) at the cost of reducing potentially important duration-specific variance in the data. In addition, by normalizing performance across different time intervals, this approach permitted us to produce an overall estimate for time perception averaged across the tested durations and to define the temporal precision of subjects’ estimates in each drug condition via the standard deviation across the four duration responses.

### fMRI image acquisition and preprocessing

A subset of 40 right-handed subjects also underwent resting state functional MRI. The remaining subjects could not be scanned due to subjects’ scheduling constraints and time available at the imaging facility. MRI scanning was conducted on a Siemens MAGNETOM Trio 3 T MR Scanner at the Henry H. Wheeler, Jr. Brain Imaging Center at the University of California, Berkeley. Anatomical images consisted of 160 slices acquired using a T1-weighted MP-RAGE protocol (TR = 2300 ms, TE = 2.98 ms, FOV = 256 mm, matrix size = 256 × 256, voxel size = 1 mm^3^). Three hundred twenty-five resting state volumes consisting of 24 transverse slices were acquired over a period of approximately 7.5 min with a gradient echoplanar imaging protocol (325 images, TR = 1370 ms, TE = 27 ms, FOV = 225 mm, matrix size = 96 × 96, voxel size = 2.3 × 2.3 × 3.5 mm).

FMRI preprocessing was performed using both the AFNI (http://afni.nimh.nih.gov) and FSL (http://www.fmrib.ox.ac.uk/fsl) software packages. Functional images were converted to 4D NIfTI format and corrected for slice-timing offsets. Motion correction was carried out using the AFNI program *3dvolreg*, with the reference volume set to the mean image of the series. Co-registration was performed with the AFNI program *3dAllineate* using the local Pearson correlation cost function optimized for fMRI-to-MRI structural alignment. The subsequent inverse transformation was used to warp the anatomical image to the functional image space. Anatomical and functional images were then normalized to a standard volume (MNI_N27: 3 mm × 3 mm × 3 mm voxels) using the FSL program *fnirt* available from the Montreal Neurological Institute (MNI; http://www.bic.mni.mcgill.ca) prior to application of connectivity analyses.

### Connectivity analysis

In order to evaluate connectivity between seed regions and other brain areas, resting state data were smoothed by a 5 mm FWHM Gaussian kernel prior to temporal bandpass filtering between 0.009 and 0.08 Hz to reduce the influence of cardiac and respiratory artifacts (Fox et al. 2005). Movement parameters and the white matter and ventricular time series, but not the global mean signal, were included as regressors of no interest. Two regions within the prefrontal cortex, the right inferior frontal gyrus (RIFG: MNI coordinates [46 7 24]) and the supplementary motor area (SMA: MNI coordinates [0 -3 59]), were then selected based upon a conversion from Talairach coordinates provided by a meta-analysis conducted by Wiener and colleagues (Wiener et al. 2010). By modeling every stereotactic coordinate as a 3D Gaussian distribution to test the probability of activation across 41 timing studies containing 446 sets of activation foci, this voxel-wise meta-analysis determined that the RIFG and SMA were the only brain regions with significant voxel activation across all timing tasks. Each ROI was defined by a set of MNI coordinates that formed the center for a sphere with 5 mm radius. A time course determined by averaging across voxels in this region was then correlated independently with every other voxel in the brain, and correlation coefficients were Fisher-transformed to allow for the application of parametric statistical tests. The so-called standard univariate contrasts were not possible due to the lack of a contrasting baseline condition and the absence of discrete task epochs. For whole brain analyses, images were normalized to the MNI template prior to the application of group-level statistics. Map-wise significance (*p* < 0.05, corrected for multiple comparisons) was determined by applying a cluster-size correction derived from the AFNI programs *3dFWHMx* and *3dClustSim* to data initially thresholded at a value of *p* < 0.005, uncorrected. Because of our hypotheses about changes in frontostriatal connectivity, the volume of a striatal mask (AAL regions 71–76 (Tzourio-Mazoyer et al. 2002)) was used to calculate the appropriate cluster-size correction of 14 voxels.

### Genetics

DNA extraction and SNP analysis were performed by the Genomics Core at the UCSF Institute for Human Genetics on salivary samples (salimetrics.com) collected during the screening visit. DNA was extracted using Gentra Puregene reagents and protocols and quantified using the Pico Green method (Molecular Probes/Invitrogen). Genotyping of the following polymorphisms via polymerase chain reaction was carried out using TaqMan® technology (Applied Biosystems): oxytocin receptor (OXTR; rs53576), oxytocin receptor (OXTR; rs2254298), dopamine D2 receptor (DRD2; rs6277), and catechol-*O*-methyltransferase (COMT; rs4680). However, in keeping with the hypothesis presented here, only the COMT rs4680 data have been analyzed. Two genetics samples could not be called due to bacterial contamination and one sample was lost at the laboratory, leading to a total of 63 samples included in statistical analysis.

### Statistical analysis

As the data set consists of both normally distributed data and data that did not meet the presumptions of normal distribution, a combination of parametric and nonparametric statistical tests were used for analysis. A Scheirer Ray Hare two-factor non-parametric ANOVA with Tukey HSD post hoc testing was used to assess differences in drug condition across time. Two-tailed Mann-Whitney tests were used to evaluate time differences between genetic samples. For the ratio approach in which performance was normalized across different time intervals, parametric tests were used. A linear mixed model evaluated the difference in the ratio (tolcapone–placebo) as a function of one fixed effect (duration) and one random effect (subject) using the Matlab function “fitlme.” Pearson’s correlation coefficients were used to calculate statistical significance of behavioral measures against drug effect. Because five behavioral questionnaires were included in comparisons based on Pearson’s correlation coefficient, we used a Bonferroni correction to allow for multiple comparisons (corrected threshold *p* value = 0.01). Parametric ANOVA and *t* test were also used to evaluate the RT data. All statistical tests were chosen in consultation with the UCSF Biostatistics Core, were performed using Excel v. 15.22 and MatLab, and were confirmed at www.Vassarstats.net.

For connectivity analyses of BOLD data, significance was calculated using statistical techniques and corrections implemented in the AFNI software package, including the functions *3dFWHMx*, *3dClustSim*, and *3dttest++.*

## Results

### Perception

**Fig. 1:**
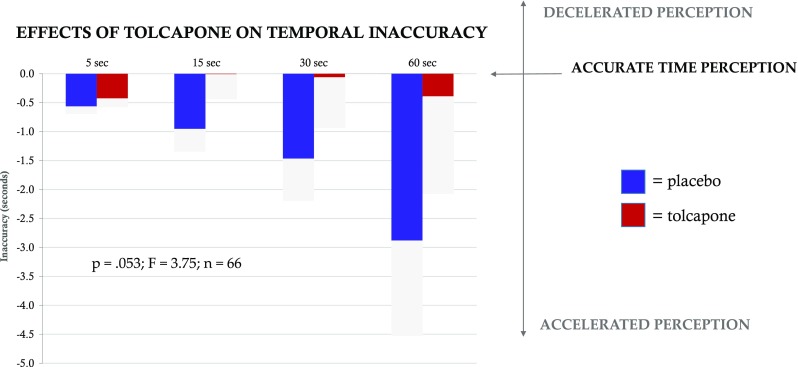
Tolcapone and temporal inaccuracy. The *x*-axis shows the four estimated temporal durations, ranging from 5 to 60 s. The *y*-axis indicates the difference in the subject’s duration estimate as compared with the desired duration (termed inaccuracy, in seconds), where 0.0 represents an exact estimate and negative numbers indicate that the subject made a key press before the goal duration had elapsed. Blue bars represent placebo, red bars represent tolcapone, and gray bars delineate standard error. A Scheirer Ray Hare two-factor non-parametric ANOVA demonstrates that compressed time perspective was significantly attenuated by administration of a single dose of tolcapone (*p* = 0.053; *F*(1) = 3.75; *n* = 66)

Following placebo administration, subjects displayed a compressed time perspective at each of the four tested time points, such that time was perceived as progressing faster than actual time. This compressed time perspective was attenuated by administration of a single dose of tolcapone and approached significance (200 mg; main effect; *p* = 0.053; *F*(1) = 3.75; *n* = 66; Fig. 1). Tukey HSD pairwise comparisons did not reveal additional significant findings at any of the four individual time points. When the data were reanalyzed using a factor of session (first visit versus second visit) rather than drug (tolcapone versus placebo), the result was not significant (*p* = 0.65 (n.s.); *F*(1) = 0.21, *n* = 66).

When subjects’ duration estimates were instead analyzed as a ratio of differences (**Δ***t*/*t*) rather than absolute differences (see “Materials and methods”) within a linear mixed model, the effect of tolcapone was significant (*T*(524) = 2.04, *p* = 0.042). There was no main effect of interval duration (*T*(524) = 0.94, *p* = 0.34 (n.s.)) and no interaction between drug and interval (*T*(524) = 0.08, *p* = 0.94 (n.s.)). Additionally, the precision of subjects’ estimates, defined as the standard deviation of the difference ratios for each of the four durations, did not differ between tolcapone and placebo conditions (difference = 0.0004 ± 0.086, *T*(65) = − 0.034, *p* = 0.97 (n.s.)). Together these data demonstrate that subjects are more accurate in their perception of time following tolcapone administration and suggest that tolcapone is acting to expand subjective time horizon.

Because tolcapone acts to inhibit the function of the dopamine-degrading enzyme COMT, we next evaluated whether the presence of a functional COMT polymorphism (Val158Met) influenced baseline time perception or response to tolcapone. COMT genotyping indicated a sample of 24 Val/Val homozygotes, 27 Met/Val heterozygotes, and 12 Met/Met homozygotes. A trend for Met homozygotes to be significantly more accurate in their baseline perception of time than Val carriers did not ultimately reach significance (*p* = 0.109; *z* = − 1.60, *n* = 63; Figure S1).

**Fig. 2:**
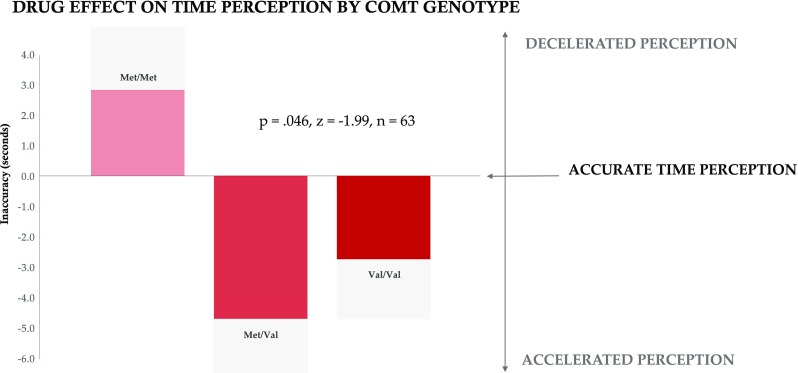
COMT genotype and the effects of tolcapone on time perception. The *x*-axis includes the three Val158Met genotypes (Met/Met, Met/Val, and Val/Val). The *y*-axis indicates inaccuracy (placebo-tolcapone), in seconds. A Mann-Whitney test demonstrated a significant difference in drug effect on time perception between COMT genotypes at 60 s (*p* = 0.046, *z* = − 1.99, *n* = 63). Gray bars delineate standard error

To explore whether these findings could be linked to behavioral and genetic measures potentially relevant to addictive and other behaviors, we assessed the relationship of accurate temporal perception at the longest duration, 60 s (see “Materials and methods”), to scores on behavioral questionnaires including the Stanford Time Perspective Inventory (STPI) and the Alcohol Use Disorders Inventory Test (AUDIT; see “Materials and methods”). Variability in baseline time perception at 60 s correlated significantly with future time perspective as measured using the STPI (*p* = 0.042, *R* = 0.25, *t* = 2.08, *n* = 66) suggesting that the effects found here could extend to future orientation. Stratification by genotype indicated that the effect of tolcapone at 60 s was carried by subjects with a Val allele, as it was this group that responded with significantly improved time perception following tolcapone administration (*p* = 0.046; *z* = − 1.99, *n* = 63; Fig. 2). This effect was not, however, seen for the 5 s duration (*p* = 0.992; *z* = 0.01; *n* = 63).

**Fig. 3:**
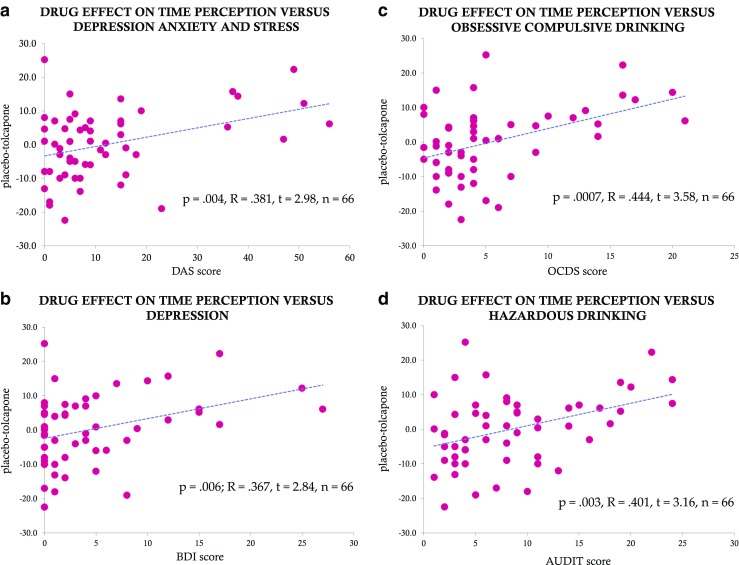
Tolcapone and behavioral/emotional measures. For all plots, the *x*-axis includes the questionnaire score, and the *y*-axis indicates the difference in the duration estimate, in seconds, for the placebo condition minus the tolcapone condition. There is a significant positive correlation (Pearson’s correlation coefficient) between drug effect on time perception at 60 s and depression, anxiety, and stress measured using the DASS (**a**, Pearson’s *p* = 0.004, *R* = 0.381, *t* = 2.98, *n* = 66) as well as a significant positive correlation between drug effect on time perception at 60 s and depression measured using the BDI (**b**, Pearson’s *p* = 0.006, *R* = 0.367, *t* = 2.84, *n* = 66). There is a significant positive correlation between drug effect on time perception at 60 s and obsessive-compulsive drinking measured using the OCDS (**c**, Pearson’s *p* = 0.0007, *R* = 0.444, *t* = 3.58, *n* = 66) as well as a significant positive correlation between drug effect on time perception at 60 s and hazardous drinking measured using the AUDIT (**d**, Pearson’s *p* = 0.003, *R* = 0.401. *t* = 3.16, *n* = 66)

In keeping with the results for future time perspective, the size of the tolcapone effect at the 60 s time perception duration also significantly positively correlated with measures of depression, anxiety, and stress, (DAS; *p* = 0.004, *R* = 0.381, *t* = 2.98, *n* = 66; BDI = *p* = 0.006; *R* = 0.367, *t* = 2.84, *n* = 66; Fig. 3a, b) and with measures of both obsessive-compulsive alcohol drinking (OCDS; *p* = 0.0007, *R* = 0.444, *t* = 3.58, *n* = 66; Fig. 3c) and hazardous alcohol consumption (AUDIT; *p* = 0.003, *R* = 0.401, *t* = 3.16; *n* = 66; Fig. 3d). No significant relationships were found for the 5 s time perspective duration. Generation of a correlation matrix demonstrated that these behavioral measures were also highly inter-correlated, with *R* values ranging from 0.45 to 0.899 (S6), arguing that they capture shared behavioral variance.

Surprisingly, the size of the tolcapone effect at 60 s did *not* correlate with impulsivity (BIS; *p* = 0.758, *R* = − 0.039, *t* = − 0.31, *n* = 66), leading us to further stratify subjects based on second-order factors within the BIS instrument (attention, motor, perseverance, self-control, cognitive instability, and cognitive complexity) and COMT genotype as well as to stratify subjects using a bi-factor model (Steinberg et al. 2013) However, further stratification did not yield significant effects.

To ensure that the effects of tolcapone on time perception were not related to non-specific motor effects or to an altered response threshold, subjects performed a control task in which they attended to a computer screen for approximately 2 min and pressed a button as quickly as possible following the repeated pseudorandom appearance of a fixation cross. To control for potential RT variations between the two testing days, we administered this task twice, once before and once approximately 3 h after drug administration. We then calculated the mean RT difference (mean RT after medication minus mean RT before medication) for both the tolcapone and placebo sessions. There was no significant difference in the mean RT difference between tolcapone and placebo (mean ± std dev = 0.7 ± 45.0 ms, *T*(52) = 0.12, *p* = 0.91 (ns). There was also no effect of drug (*T*(51) = − 1.15, *p* = 0.26) and no effect of session (*T*(51) = 1.1, *p* = 0.28) on RT and no influence of genotype on either the effect of drug (*F*(2,51) = 0.14, *p* = 0.87) or the effect of session (*F*(2,51) = 0.09, *p* = 0.91).

### fMRI

**Fig. 4:**
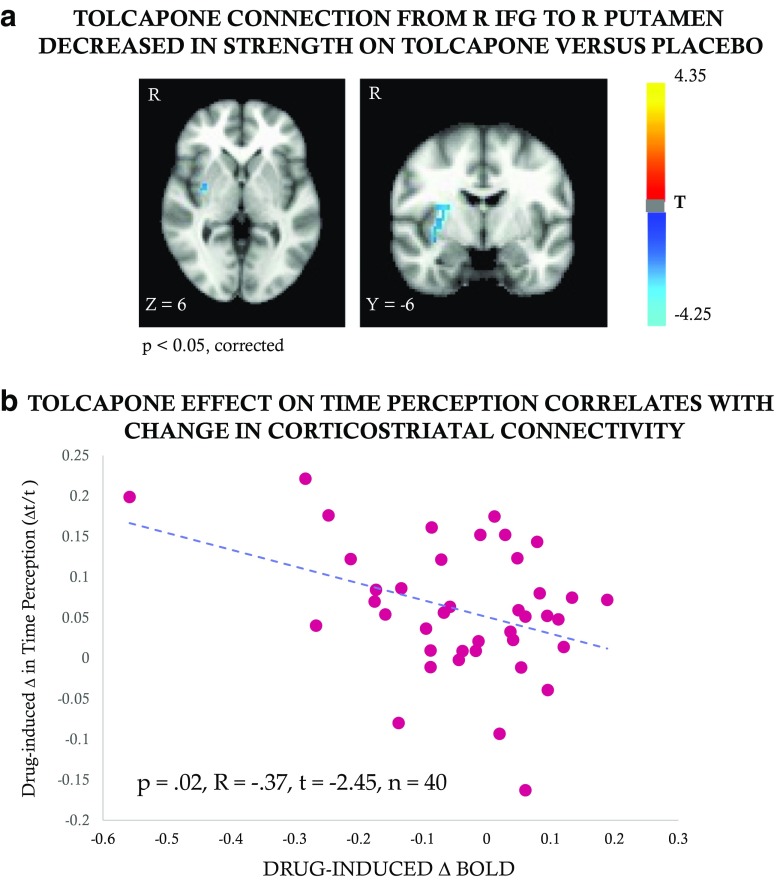
Connectivity and time perception. **a** The connection from RIFG to the right putamen decreased in strength on tolcapone versus placebo across all subjects, as indicated by the cool/blue color (*p* < 0.05, corrected; R putamen cluster (cluster size = 16) with MNI coordinates *x* = 33, *y* = 6, *z* = 5; peak *T* value = 3.63; peak *p* value = 0.00081). **b** The strength of the decrease in corticostriatal connectivity between the RIFG and R putamen (*x*-axis) correlated inversely with the increase in duration estimation (*y*-axis) on tolcapone versus placebo (Pearson’s *p* = 0.02, *R* = − 0.37, *t* = − 2.45, *n* = 40)

A seminal meta-analysis of time perception (Wiener et al. 2010) identified the right inferior frontal gyrus (RIFG) and supplementary motor area (SMA) as the two brain regions activated in response to a variety of timing conditions across a number of published motor and perceptual tasks. Using these two areas as seed regions and assuming a basal ganglia mask to assess our hypothesis regarding changes in corticostriatal connectivity, we found that a connection from RIFG to the right putamen significantly decreased in strength on tolcapone versus placebo across all subjects (Fig. 4a, *p* < 0.05, corrected; R putamen cluster (cluster size = 16) with MNI coordinates *x* = 33, *y* = 6, *z* = 5; peak *T* value = 3.63; peak *p* value = 0.00081). No other significant findings were seen when we expanded our search to the rest of the brain, and no significant results were seen for the SMA seed (data not shown). To assess whether the magnitude of the change in strength of this connection covaried with our behavioral results, we used the ratio of the change in time perception to the desired duration, **Δ***t*/*t* (see “Materials and Methods”), to compute a mean change value for each subject across all four durations. Intriguingly, the magnitude of the decrease in corticostriatal connectivity between the RIFG and R putamen correlated inversely with the increase in duration estimation on tolcapone versus placebo (*p* = 0.02, *R* = − 0.37, bootstrapped confidence interval [− 0.59, − 0.023], *t* = − 2.45, *n* = 40; Fig. 4b). These data remained significant when analyzed using a non-parametric test to reduce the influence of individual data points (Kendall’s tau = − 0.185, *p* = 0.047).

## Discussion

We have previously demonstrated that the COMT inhibitor tolcapone reduces impulsive decision-making in a delay discounting paradigm (Kayser et al. 2012). Consistent with the hypothesized role of dopamine in component processes related to impulsivity and brain circuitry, we now show that compressed time perspective can be ameliorated by a single dose of the COMT inhibitor tolcapone, and that these effects cannot be explained by session effects or changes in low-level motor responding. Moreover, we found a significant positive correlation between temporal accuracy at a short time interval (specifically, 60 s) and a more protracted measure of future orientation, suggesting both that the measures of time perception collected here may indeed be pertinent to a far longer time horizon (see Grondin 2010), and that augmenting frontal dopamine tone could be useful in enabling vulnerable subject populations to develop a more accurate sense of time.

These findings are consistent with previous work that evaluated the influence of dopamine on time perception. In keeping with our current results demonstrating the possible benefit of increasing frontal dopamine tone, a past study found that dopamine depletion in healthy subjects impaired the accuracy of time perception, most likely by attenuating neural activity in the supplementary motor area and putamen that is hypothesized to be important for initiating storage of temporal information into working memory (Coull et al. 2012). Similarly, the seminal behavioral work of Rammsayer (Coull et al. 2011; Rammsayer 1997a, 1997b) demonstrated that D2-receptor antagonists impair interval discrimination at sub- and suprasecond durations. Notably, no effects of L-dopa, a dopamine precursor, on duration discrimination were seen (Rammsayer 1989a, 1989b), emphasizing the potential importance of the brain region in which increased dopamine tone is occurring (diffuse in response to L-dopa, more specifically cortical/frontal with tolcapone). Taken together, these data suggest that individuals who are functionally hypodopaminergic at baseline have an attenuated time horizon and impaired time perception that might be improved by increasing dopamine availability in frontal regions.

The passage of time is typically underestimated both in healthy control subjects and in patient populations (Buhusi and Meck 2005; Harrington et al. 1998; Peterburs et al. 2013). Our current data are consistent with the Vierordt effect, which describes a baseline tendency to underestimate time durations longer than approximately 5 s (Bausenhart et al. 2014; Peterburs et al. 2013; Shi et al. 2013). Taken together, the present results extend the abovementioned findings by demonstrating that increasing frontal tone can improve time perception, and they suggest that the potential of tolcapone to generate more accurate time perception in some subjects might be mediated by a reduction in the perceived “slowness” of time.

Our results are also consistent with past work evaluating the effect of polymorphisms in dopamine-related genes. In keeping with a myriad of previous reports related to impulsivity and executive control (e.g., Grant et al. 2015; Schacht 2016), the data presented here suggest that a genetic polymorphism in the protein target of tolcapone, COMT, may contribute to altered time perception. Recent findings have demonstrated, for example, that COMT Val158Met Val/Val homozygotes express greater variability on a suprasecond timing assessment (Wiener et al. 2011). A subsequent study by this same group showed that Met carriers displayed reduced memory variability in this same suprasecond time window (Balci et al. 2013). Our current data suggest that individuals with at least one copy of the Val allele are more likely to exhibit a compressed time horizon at baseline and to display the most obvious improvement in time perception following tolcapone administration. However, because of the small sample size and limited power of the current dataset, additional studies are necessary to fully assess the effects of tolcapone by genotype.

Considering the above pharmacological and genetic data, the cognitive mechanisms by which tolcapone may improve time perception are most likely located in cortical and corticostriatal circuitry. Importantly, the results of the studies described in the preceding paragraphs occurred primarily at suprasecond as opposed to subsecond durations, a time frame that is thought to engage cortically based cognitive processes. Based on these data, it has been proposed that increases in cortical dopamine tone might reduce memory variance and increase the fidelity of working memory encoding (Balci et al. 2013; Coull et al. 2012). In addition, dopamine denotes events as salient (Berridge and Robinson 1998; Robinson and Berridge 1993; Salamone and Correa 2012) and is necessary for both assigning credit to available choices (Cockburn et al. 2014) and facilitating action selection (Bogacz et al. 2007). By influencing a connection between RIFG and striatum, dopamine might initiate a working memory trace that can be used as a time stamp for credit assignment and for encoding and remembering credit assignment to facilitate future action selection. Perhaps by robustly marking an event as salient, dopamine increases the depth of temporal and motivational encoding via the RIFG and encourages the memory of these salient events over longer time durations.

That tolcapone led to a decrease, rather than an increase, in connectivity between the RIFG and the putamen deserves further consideration. At least two possibilities exist. First, a nearby, potentially overlapping, region of the RIFG has been shown to play a pivotal role in inhibitory control; specifically, it is commonly activated when subjects perform tasks requiring intermittent unexpected inhibition of planned actions, as in the stop signal paradigm (reviewed in Aron et al. 2014). While controversy continues to exist about the cognitive process instantiated by this region—i.e., whether it implements an inhibitory “stop signal” itself or whether it is part of a larger network that provides a more general monitoring function (Hampshire and Sharp 2015)—attenuation of its connectivity with the putamen may reduce the pressure to stop the temporal estimation process, thereby permitting a longer duration time estimate. Alternatively, because of the inverted U-shaped response to dopamine tone in many frontal regions (Cools and D'Esposito 2011), it is possible that decreased connectivity between RIFG and putamen reflects a decline in attention related to too much, rather than too little, dopamine tone. Because reductions in attention are known to prolong duration estimates (Coull et al. 2004; Mangels et al. 1998), tolcapone’s action may represent an adverse effect that nonetheless appears as a behavioral improvement. This hypothesis would be consistent with data showing that impaired connectivity between the IFG and striatum is associated with executive dysfunction (Quan et al. 2013).

If this latter possibility is true, then a decline in working memory or a change in a related function, counting, would not easily explain our other results with tolcapone, which include reductions in the discounting of delayed rewards (Kayser et al. 2012), increases in goal-directed exploration (Kayser et al. 2015), and more altruistic distribution of resources (Saez et al. 2015). Moreover, we did not see changes in a control task that required sustained attention for the intermittent appearance of a fixation cross. While we were unable to directly control for the use of subvocal rehearsal (such as counting) as a strategy to assist subjects in assessing the passage of time, previous research has shown that counting does not improve accuracy within the temporal window used in our time perception task (Hinton et al. 2004; Thönes and Hecht 2017). Nonetheless, addressing these hypotheses would clearly benefit from future experiments to directly evaluate task-related (as opposed to resting state) neural changes, as well as relevant tests of working memory.

Irrespective of the underlying cognitive mechanisms, the changes we observed within the corticostriatal circuitry that processes temporal information correlated with scores on instruments that assess behaviors associated with changes in dopamine functioning. The current data show, for example, that the effects of tolcapone on temporal impulsivity may be most beneficial in those with affective disorders, as evaluated by questionnaires addressing stress, anxiety, and depression and comorbid alcohol use disorder (AUD). Significant relationships were seen with both the AUDIT and obsessive-compulsive drinking scales at the 60 s time duration, raising the possibility that tolcapone might contribute in particular to the treatment of AUD. However, contrary to our initial hypothesis, the size of the tolcapone effect on time perception did not correlate with impulsivity as measured by the Barratt Impulsiveness Score (BIS). Because we previously reported greater levels of impulsivity in individuals with alcohol use disorder (Mitchell et al. 2005; Mitchell et al. 2007), we decided to further classify the current subject population based on their level of alcohol consumption (median split by AUDIT score), as it is possible that the relationship between impulsivity and drinking that we previously reported in subjects with AUDs would not be present in low drinking subjects. Testing homogeneity of the two resulting regressions indicated a trend towards significance (*p* = 0.057, *F* = 3.77, *n* = 66), suggesting that there may be a difference in the relationship between impulsivity and the effect of tolcapone on time perception in hazardous drinkers as compared to those who drink within the normal range. It is therefore possible that this difference in subgroups blunted our ability to appropriately study the relationship between impulsivity and the effects of a COMT inhibitor on time perception.

The present data were collected from a group of young, generally healthy, and ethnically diverse subjects who had volunteered to participate in neuroimaging and pharmacology studies in a university setting. As such, these data may not completely characterize the population at large, nor be indicative of temporal processing in an older group of individuals. Additional studies would be needed to determine how the effects of frontal dopamine on time perception change as we age. More broadly, as additional behavioral and neurological factors emerge that predict the temporal effects of increased frontal dopamine tone, these can be used to characterize groups that will most benefit from administration of a COMT antagonist.

In summary, previous research indicates that lower frontal dopamine levels result in poorer executive control (Boettiger et al. 2007; Gallagher et al. 2015) and difficulties with impulsivity and time perception (Blum et al. 2007; Gold et al. 2015). Our current findings suggest that compressed time perception, a hypothesized component of impulsivity (Peters and Buchel 2011), can be improved by administration of the COMT inhibitor tolcapone, perhaps by decreasing connectivity between RIFG and R putamen. If replicated, the data reported here could be relevant to individuals suffering from a number of clinical conditions that are characterized by deficits in time perception. Further studies could then determine whether COMT inhibitors might be therapeutically effective in treating psychiatric conditions, such as alcohol and substance abuse, that are linked to perturbations in time perception and decision-making.

## Electronic supplementary material

ESM 1 (PPTX 638 kb)
