# Supplementary material for: Dopamine, time perception, and future time perspective
Source: Psychopharmacology (Berl). 2018 Jul 19;235(10):2783–93. doi: 10.1007/s00213-018-4971-z (PMC6182591; doi:10.1007/s00213-018-4971-z)
Supplement: Supplementary file 1 — (PPTX 638 kb) [file 213_2018_4971_MOESM1_ESM.pptx]

## Slide 1
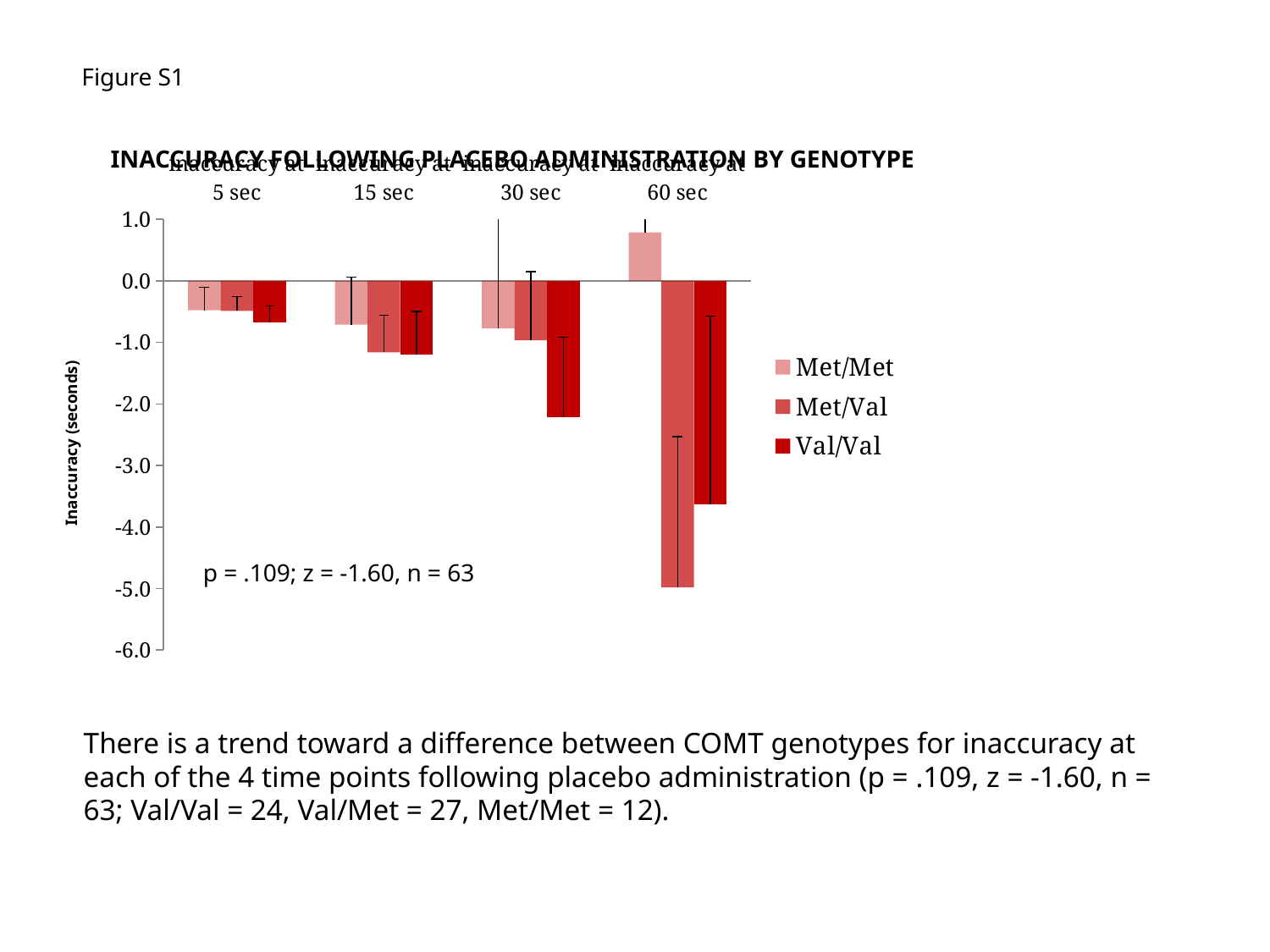

Figure S1
### Chart:
| Category | | | |
|---|---|---|---|
| inaccuracy at 5 sec | -0.476090909090909 | -0.487814814814815 | -0.669565217391304 |
| inaccuracy at 15 sec | -0.712636363636364 | -1.161185185185185 | -1.203260869565217 |
| inaccuracy at 30 sec | -0.771909090909091 | -0.966555555555555 | -2.222217391304347 |
| inaccuracy at 60 sec | 0.786454545454546 | -4.982259259259258 | -3.631130434782609 |INACCURACY FOLLOWING PLACEBO ADMINISTRATION BY GENOTYPE
Inaccuracy (seconds)
p = .109; z = -1.60, n = 63
There is a trend toward a difference between COMT genotypes for inaccuracy at each of the 4 time points following placebo administration (p = .109, z = -1.60, n = 63; Val/Val = 24, Val/Met = 27, Met/Met = 12).

## Slide 2
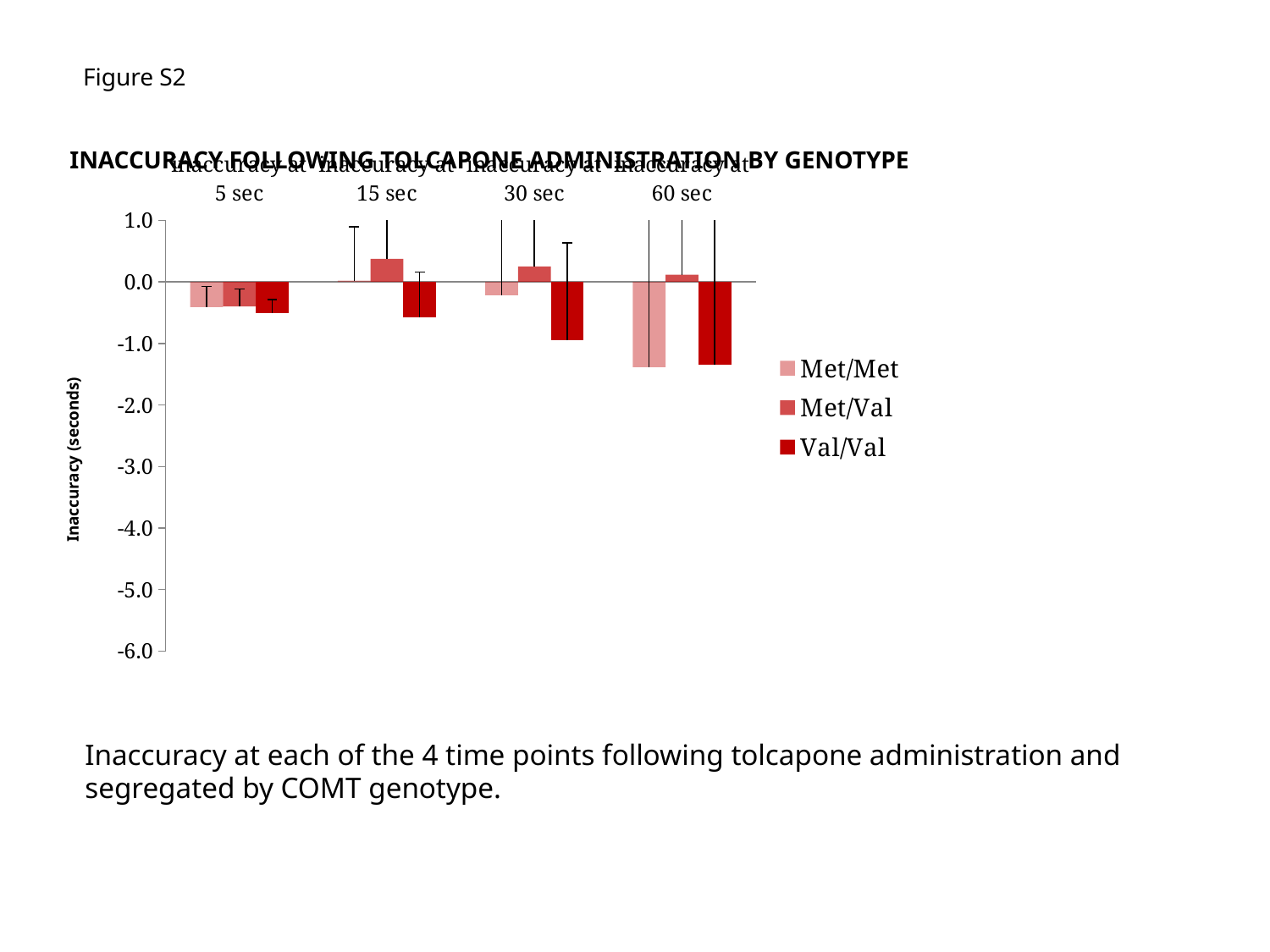

Figure S2
INACCURACY FOLLOWING TOLCAPONE ADMINISTRATION BY GENOTYPE
### Chart:
| Category | | | |
|---|---|---|---|
| inaccuracy at 5 sec | -0.406818181818182 | -0.396 | -0.509434782608696 |
| inaccuracy at 15 sec | 0.0199090909090907 | 0.374925925925926 | -0.576260869565217 |
| inaccuracy at 30 sec | -0.214636363636364 | 0.252259259259259 | -0.949478260869565 |
| inaccuracy at 60 sec | -1.384727272727273 | 0.118185185185185 | -1.344565217391304 |Inaccuracy (seconds)
Inaccuracy at each of the 4 time points following tolcapone administration and segregated by COMT genotype.

## Slide 3
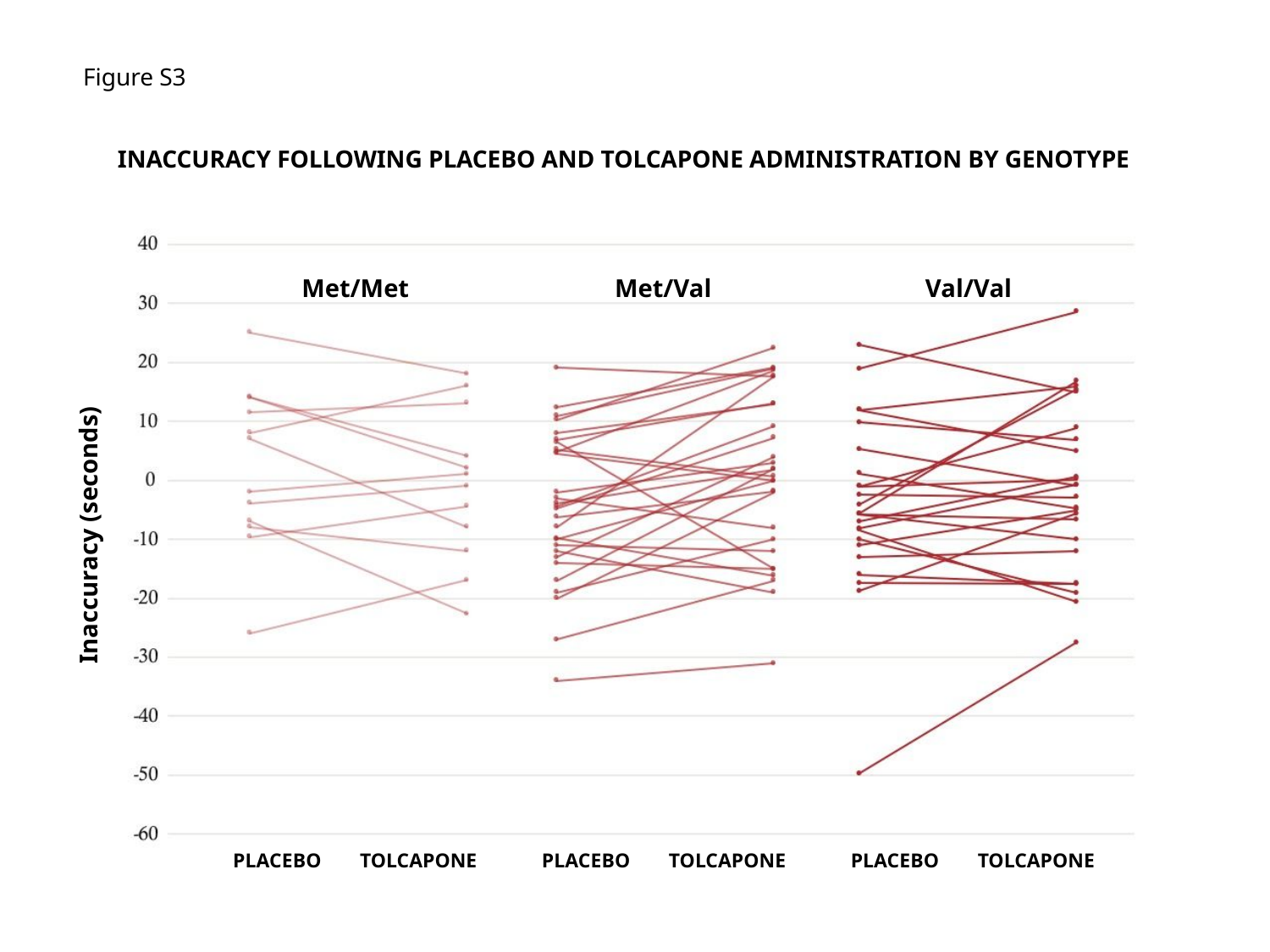

Figure S3
INACCURACY FOLLOWING PLACEBO AND TOLCAPONE ADMINISTRATION BY GENOTYPE
Met/Val
Val/Val
Met/Met
PLACEBO	TOLCAPONE
PLACEBO	TOLCAPONE
PLACEBO	TOLCAPONE
Inaccuracy (seconds)

## Slide 4
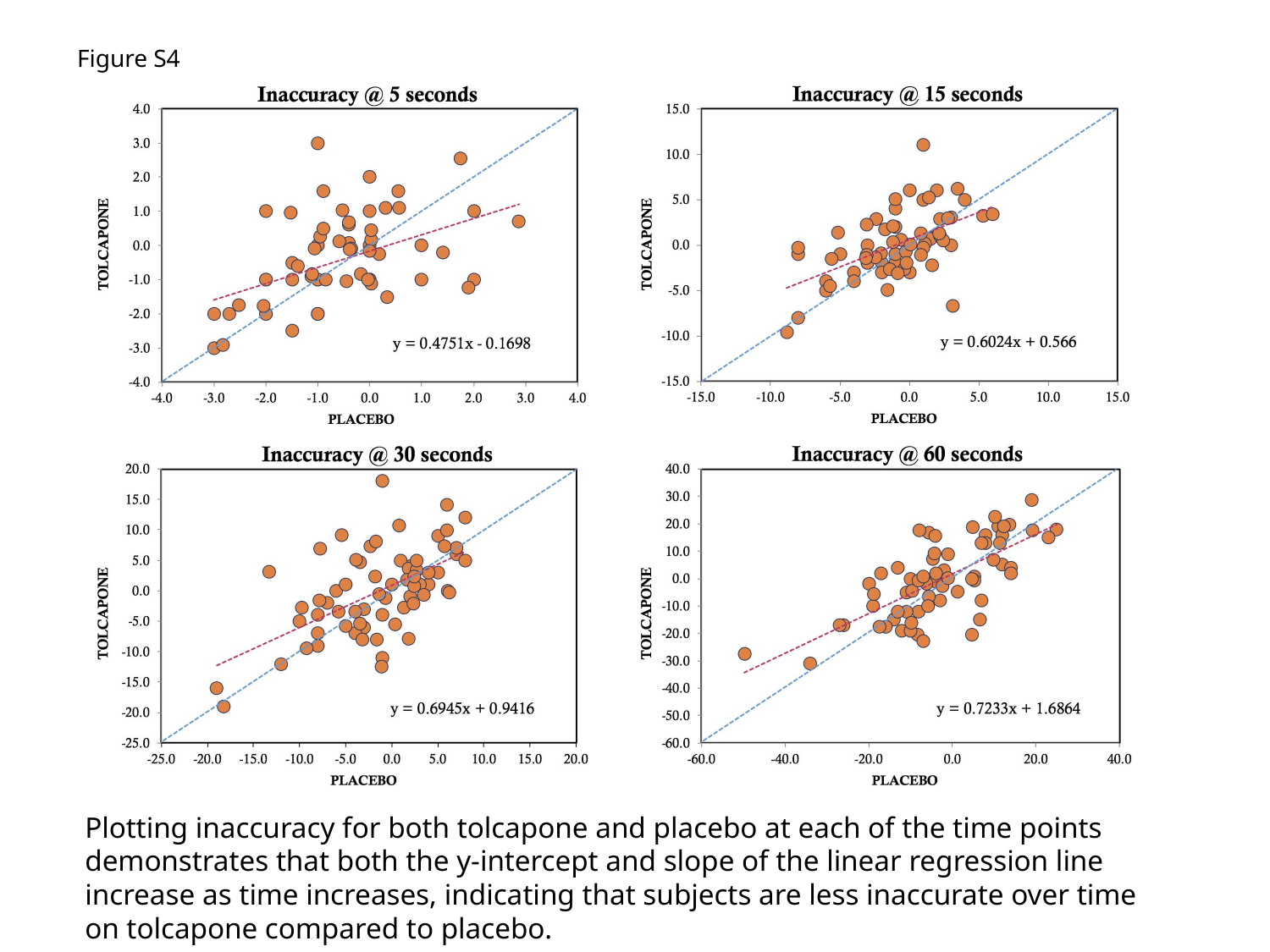

Figure S4
Plotting inaccuracy for both tolcapone and placebo at each of the time points demonstrates that both the y-intercept and slope of the linear regression line increase as time increases, indicating that subjects are less inaccurate over time on tolcapone compared to placebo.

## Slide 5
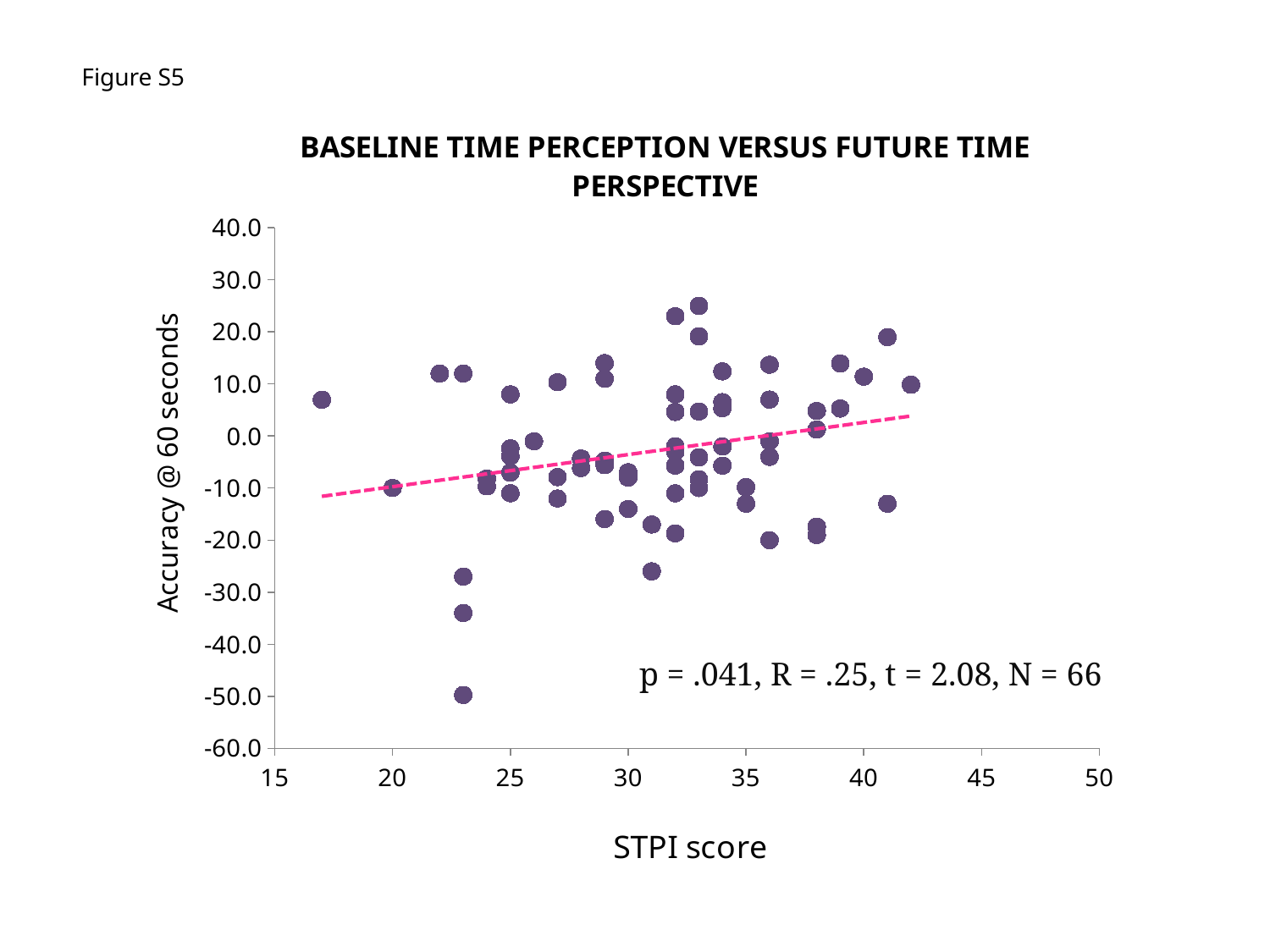

Figure S5
### Chart: BASELINE TIME PERCEPTION VERSUS FUTURE TIME PERSPECTIVE
| Category | |
|---|---|p = .041, R = .25, t = 2.08, N = 66

## Slide 6
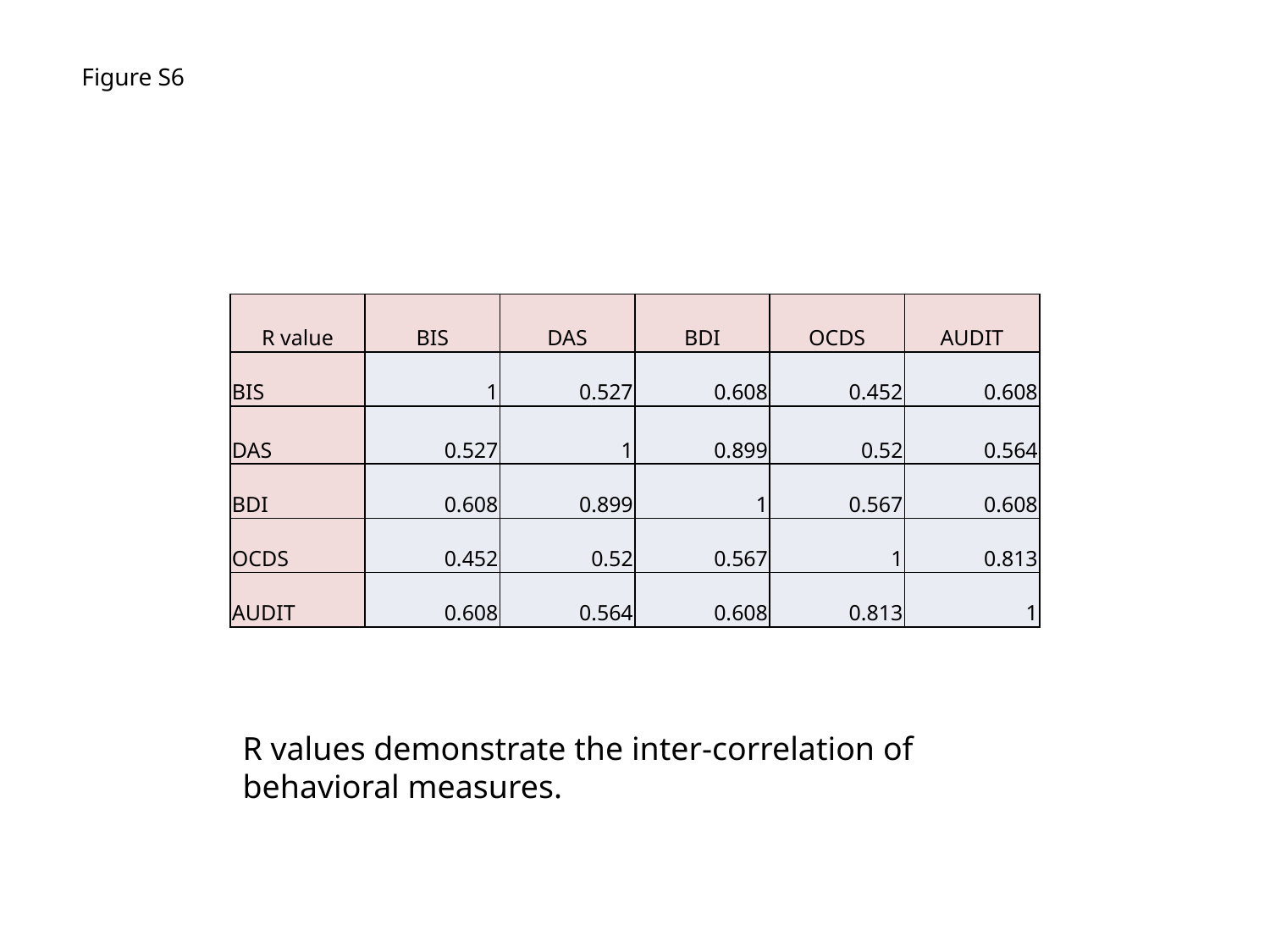

Figure S6
| R value | BIS | DAS | BDI | OCDS | AUDIT |
| --- | --- | --- | --- | --- | --- |
| BIS | 1 | 0.527 | 0.608 | 0.452 | 0.608 |
| DAS | 0.527 | 1 | 0.899 | 0.52 | 0.564 |
| BDI | 0.608 | 0.899 | 1 | 0.567 | 0.608 |
| OCDS | 0.452 | 0.52 | 0.567 | 1 | 0.813 |
| AUDIT | 0.608 | 0.564 | 0.608 | 0.813 | 1 |
R values demonstrate the inter-correlation of behavioral measures.
